# Optical clearing and fluorescence deep-tissue imaging for 3D quantitative analysis of the brain tumor microenvironment

**DOI:** 10.1007/s10456-017-9565-6

**Published:** 2017-07-11

**Authors:** Tonny Lagerweij, Sophie A. Dusoswa, Adrian Negrean, Esther M. L. Hendrikx, Helga E. de Vries, Jeroen Kole, Juan J. Garcia-Vallejo, Huibert D. Mansvelder, W. Peter Vandertop, David P. Noske, Bakhos A. Tannous, René J. P. Musters, Yvette van Kooyk, Pieter Wesseling, Xi Wen Zhao, Thomas Wurdinger

**Affiliations:** 1 Neuro-oncology Research Group, VU University Medical Center, CCA Room 3.60, De Boelelaan 1117, 1081 HV Amsterdam, The Netherlands; 2 Brain Tumor Center Amsterdam, VU University Medical Center, Amsterdam, The Netherlands; 3 Department of Neurosurgery, VU University Medical Center, Amsterdam, The Netherlands; 4 Department of Molecular Cell Biology and Immunology, VU University Medical Center, Amsterdam, The Netherlands; 5 Department of Physiology, VU University Medical Center, Amsterdam, The Netherlands; 6 Department of Pathology, VU University Medical Center, Amsterdam, The Netherlands; 7 Integrative Neurophysiology, Centre for Neurogenomics and Cognitive Research, VU University, Amsterdam, The Netherlands; 8 Princess Máxima Center for Pediatric Oncology, University Medical Center Utrecht, Utrecht, The Netherlands; 9 Department of Neurology, Massachusetts General Hospital, Harvard Medical School, Boston, MA USA

**Keywords:** Vasculature, Imaging, 3D, CLARITY, iDISCO, Multicellular network

## Abstract

**Background:**

Three-dimensional visualization of the brain vasculature and its interactions with surrounding cells may shed light on diseases where aberrant microvascular organization is involved, including glioblastoma (GBM). Intravital confocal imaging allows 3D visualization of microvascular structures and migration of cells in the brain of mice, however, with limited imaging depth. To enable comprehensive analysis of GBM and the brain microenvironment, in-depth 3D imaging methods are needed. Here, we employed methods for optical tissue clearing prior to 3D microscopy to visualize the brain microvasculature and routes of invasion of GBM cells.

**Methods:**

We present a workflow for *ex vivo* imaging of optically cleared brain tumor tissues and subsequent computational modeling. This workflow was used for quantification of the microvasculature in relation to nuclear or cellular density in healthy mouse brain tissues and in human orthotopic, infiltrative GBM8 and E98 glioblastoma models.

**Results:**

*Ex vivo* cleared mouse brain tissues had a >10-fold imaging depth as compared to intravital imaging of mouse brain in vivo. Imaging of optically cleared brain tissue allowed quantification of the 3D microvascular characteristics in healthy mouse brains and in tissues with diffuse, infiltrative growing GBM8 brain tumors. Detailed 3D visualization revealed the organization of tumor cells relative to the vasculature, in both gray matter and white matter regions, and patterns of multicellular GBM networks collectively invading the brain parenchyma.

**Conclusions:**

Optical tissue clearing opens new avenues for combined quantitative and 3D microscopic analysis of the topographical relationship between GBM cells and their microenvironment.

**Electronic supplementary material:**

The online version of this article (doi:10.1007/s10456-017-9565-6) contains supplementary material, which is available to authorized users.

## Introduction

Glioblastomas (GBMs) remain incurable, partly because of extensive, diffuse infiltration of the GBM cells into their surrounding microenvironment. GBM cell invasion and proliferation leads to changes in the microvasculature, tissue perfusion, and brain architecture. Increased awareness of spatial heterogeneity of the GBM cells, in relation to the microvasculature, and intervascular tissue microenvironment [1–4], has raised the need for 3D analyses of brain tumor tissues.

Optical 3D analysis allows imaging of brain structures at cellular resolution and may serve as a bridge between CT, PET, or MRI and classical microscopic histology and immunohistochemistry [5, 6]. Intravital confocal microscopy enables 3D fluorescence imaging on a cellular level [7, 8], but its use is hampered by sedation time of the animal, limited imaging depth, small field of view, and limitations associated with fluorescent labeling [8]. These limitations do not apply to *ex vivo* optical imaging. For a long time, optical imaging of 3D structures was dependent on histological sectioning [3, 9, 10]. This sectioning is, however, a laborious and challenging task, since at least several dozens of histological slices have to be obtained and properly aligned for the creation of an informative 3D image. To avoid these laborious and error-prone approaches, optical slicing methods were developed. Optical slicing involves clearing of tissues to make them transparent, thus enabling deep-tissue fluorophore excitation and detection. Although optical clearing techniques were described already more than a century ago [11], the interest in this approach was boosted by the development of more advanced clearing techniques such as 3DISCO/iDISCO/uDISCO, *Scale,* SeeDB, and CLARITY [12–20], which all have their specific advantages and disadvantages [18, 21]. Besides new clearing techniques, other major contributions to optical slicing methods were the development and improvement of equipment such as multi-photon microscopes and light sheet microscopes. Furthermore, numerous relevant software tools have been developed, including ImageJ, Vaa3D, Farsight, NeuronStudio, Amira, and Imaris [22–25].

Here, we employed optical clearance methods to study GBM cells in the mouse brain microenvironment. We demonstrate that optically cleared tissues can be imaged up to at least 2000 μm depth, at subcellular resolution. This allowed detailed 3D visualization of the brain tumor microenvironment and revealed patterns of networks of collectively invading GBM cells.

## Methods

### Animal care guidelines

All animal experiments were approved by the VU University Medical Center Animal Welfare review board. Female, specific pathogen-free, athymic nude-Foxn1^nu^ mice (6–8 weeks; Harlan/Envigo, The Netherlands) were kept in filter top cages and received food and water ad libitum.

### Intravital confocal imaging

Application of a cranial window for intravital imaging of the mouse brain was based on the method as described by Mostany et al. [26]. Three mice were anesthetized by isoflurane inhalation and received temgesic (0.05 mg/kg) preoperatively and dexamethasone (0.2 mg/kg) with carprufen (5 mg/kg) postoperatively to prevent edema. With a 0.8-mm cutter, an area with a diameter of 5 mm in the skull was opened at the designated location. After hemostasis, a drop of silicon oil was placed onto the dura and a glass coverslip was glued on top of the craniotomy. Blood vessels were fluorescently stained by intravenous injection of Lycopersiconesculentum (*tomato)* lectin^tomato^-FITC. Images with a diameter of 350 µm were captured at 50-µm depth intervals.

### Orthotopic GBM xenograft models

Human GBM8 glioblastoma cells [27] and human E98 glioblastoma cells were lentivirally transduced with a lentivirus vector to stably express the mCherry fluorescent protein and firefly luciferase (Fluc) [28]. GBM8-FM cells were cultured as neurospheres in serum-free medium, supplemented with growth factors (2% of B27 supplement, 1% of N2 supplement, 2 µg/ml heparin, 20 ng/ml recombinant human EGF, 10 ng/ml recombinant human bFGF). E98-FM cells were injected subcutaneously in a donor mouse. When the tumor reached a diameter of 1 cm, the tumor was removed and a single-cell suspension was prepared. The harvested GBM cells were washed once with PBS and concentrated by centrifugation to a concentration of 1 × 10^5^ cells per µl. Mice were stereotactically injected with 5 × 10^5^ tumor cells into the striatum. Intracranial injections were performed under isoflurane anesthesia and systemic and topical analgesia (buprenorphine, 0.1 mg/kg; lidocaine 0.5%). The coordinates used for injections were 0.5 mm *X*, 2 mm *Y*, and −3 mm *Z* from the bregma [29]. Tumor progression was confirmed by Fluc in vivo bioluminescence imaging (BLI) after i.p. injection of d-luciferin (100 mg/kg) and acquiring the photon flux (p/s) using the Xenogen-IVIS Lumina system under isoflurane anesthesia.

### Photoacoustic imaging (PAI)

Three mice with GBM8-FM tumors were used to evaluate photoacoustic imaging. PAI was performed on a Vevo LAZR imaging station (FUJIFILM Visualsonics Inc. Toronto, ON, Canada) which features a hybrid ultrasound and photoacoustic transducer with a tunable nanosecond pulsed laser. Photoacoustic oxygenation signals were collected at 750 and 850 nm and with the provided FUJIFILM Visualsonics imaging analysis software the average oxygen saturation of the hemoglobulin was calculated and visualized [30].

### CLARITY optical clearing and immunolabeling

Mice were perfused with CLARITY hydrogel according to the protocol described by Chung et al. [15]. Animals were deeply anesthetized with an overdose of ketamin/xylazin and perfused with ice-cold PBS to remove highly auto-fluorescent hemoglobin, followed by perfusion with hydrogel solution (4% PFA, 4% acrylamide, 0.05% bis-acrylamide, 0.25% VA044 initiator in PBS). Brains were incubated for 24–72 h in hydrogel at 4 °C, and polymerization of the hydrogel was accomplished by deoxygenation, followed by thermal initiation of the polymerization reaction. Polymerized mouse brains were cut into 3–5 mm slices and washed to remove free monomers and PFA. An electrophoretic tissue chamber (ETC) was made, based on reported specifications [31] (Supplementary Figure S2A). SDS-clearing buffer (200 mM sodium borate, 4% SDS, pH8.5) was kept at 37 °C and pumped through the ETC chamber at a speed of 0.6–1.2 l/min. Tissues were electrophorized during 3–5 days at a constant voltage of 20 V. The SDS-clearing solution was replaced if pH dropped below pH 8. After electrophoretic lipid removal, tissues were washed for 24 h in PBS containing 0.1% Triton X100 at 37 °C. Tissue samples were stained, depending on their sizes, for 1–7 days in PBS containing DAPI and lectin^tomato^-DyLight488 or lectin^tomato^-DyLight594 (Vector Laboratories, dilution 1:500) or lectin^tomato^-FITC (Vector Laboratories, dilution 1:500). Subsequently, samples were washed for 24 h in PBS and finally incubated for 6–24 h in 80% glycerol to match refractive indices, resulting in transparency of the samples.

### iDISCO immunolabeling and optical clearing

Brains of mice with established tumor progression were fixed and stored in 4% buffered formalin for at least 24 h, up to more than 2 years. These brains were sliced into 3–5 mm thick sections and processed for immunolabeling and optical clearing with the methanol iDISCO procedure [32]. Samples were permeabilized with increasing methanol concentrations to a final concentration of 100% methanol. Next, the brain sections were bleached overnight at 4 °C in hydrogen peroxide/methanol (ratio of 1:5), after which tissues were gradually rehydrated to PBS and washed in PBS with 0.2% Triton X100. To block residual binding sides, tissues were incubated for three days at room temperature (RT) with antibody diluent (DAKO). Following blocking, the samples were stained for seven days with primary antibody (anti-RFP, which binds the mCherry reporter protein expressed in tumor cells) and DyLight488-conjugated lectin^tomato^ at RT. Excess primary antibodies or lectins were washed away during 3 days. Tissues were incubated with the secondary antibody (AlexaFluor594-conjugated anti-rabbit) for seven days. Unbound antibody was washed away with PBS–Triton during three days, after which tissues were dehydrated by gradually increasing methanol concentrations to a final solution of 100% methanol. Next, remaining lipids were removed by incubation in dichloromethane (DCM)/methanol and 1-h incubation with 100% DCM. To make the samples transparent, samples were incubated in dibenzyl ether (DBE) for refractive index matching. It is important to use fresh DCM and DBE as oxidation of these reagents may result in suboptimal clearing and a brownish color of the tissues. Transparent samples were stored in the dark in a closed vial completely filled with DBE until image acquisition for a period up to 6 months.

### 3D image acquisition

Intravital imaging was performed on a custom-built two-photon laser scanning microscope using Olympus objectives [33]. *Ex vivo* two-photon images were captured on a LaVision BioTec microscope system, based on an Olympus BX51WI microscope, connected to a pulsed tunable (740–1070 nm) laser (Chameleon, Coherent). CLARITY-cleared tissues were mounted in an imaging chamber filled with 80% glycerol, whereas iDISCO-cleared tissues were mounted in a flexible imaging chamber, created with adhesive poster pads, which was filled with DBE (Supplementary Fig. 3B). Images were acquired with a water immersion objective (20× magnification, NA 1.00, maximum working distance 2000 µm) at a voxel size of 0.54 × 0.54 × 1–5 µm. Epifluorescence signals were separated by dichroic mirrors and filters (DAPI: 420/50, FITC/DyLight488: 525/50) and collected by a photomultiplier tube (PMT). Laser power was set with an automatic laser power increment, which increased laser power gradually from 5 to 20% over a depth of maximum 2000 µm. Lectin^tomato^-labeled microvasculature and DAPI-stained nuclei were imaged at an excitation wavelength of 800 nm, which simultaneously excites all fluorophores. Alternatively, images were acquired with a Leica SP8 confocal microscope, equipped with a pulsed white light laser and a 10 × 0.4NA air objective or a Nikon A1R confocal microscope with a 20 × 0.8NA air objective. These images were taken with a voxel size of 1 µm^3^, and fluorescent signals were recorded using gated hybrid detectors.

### Data processing

Image reconstruction of the 2-photon images was performed using FIJI with the ‘Stitch Sequence of Grids of Images’ plugin of FIJI [34]. Background subtraction was performed by application of a Gaussian filter. Images obtained with the other microscopes were imported directly into Imaris software (version 7, Bitplane) and stitched automatically.

### Quantification of signal intensity

Quantification of signal intensity was performed using FIJI. Epifluorescent signal intensity as a function of imaging depth was determined by measurement of median signal intensity at all imaging depths. Median fluorescence was normalized to the intensity of the most superficial slide.

### 3D modeling of the vasculature and cell density

Fluorescently stained vessel surfaces were detected and converted into a mask. This mask was used to create a new, binary, channel in which the voxels outside the mask were set to zero and inside the mask to a fixed value. This binary mask was used to trace blood vessels by the ‘Filaments Tracer’ option of Imaris, which is a tool developed for the automatic detection of filament-like structures. Although this ‘Filaments Tracer’ tool is not completely compatible for the detection of vessel structures, as it does not allow the formation of loops, the detected structures co-localized with most of the fluorescently labeled vessels. During the detection process, vascular modeling required assignment of minimal (2 µm) and maximal (10 µm) vessel diameter, and manual definition of the thresholds for starting points and seeding points. Nuclei or RFP-positive cells were rendered using the ‘Spots Rendering’ application of Imaris. This application visualized the nuclei or cells as artificial solid objects, enabling quantification of number of nuclei per ROI and distance of the cells to the nearest blood vessel. All computational modeling was done ‘unbiased,’ e.g., without additional improvement of the modeling by manual deletion or insertion of vessel parts.

### 2D immunohistochemistry

Sections of 5-µm FFPE tumor-containing brain tissues were processed after heat-induced antigen retrieval with Tris–EDTA (pH 9.0). Sections were stained with DyLight488-labeled lectin^tomato^ together with rabbit anti-CD31 antibody (Abcam, ab 28364; dilution 1:1000) or rabbit anti-RFP antibody (Tebu-Bio, cat. no. 600-901-397; dilution 1:500). The rabbit antibodies were detected with AlexaFluor594-labeled anti-rabbit as secondary antibody. Alternatively, slides were stained with hematoxylin/eosin. Image acquisition was done with a Zeiss Axio Scan Z1 slide scanner.

### Statistical analysis

Statistical analyses were performed using GraphPad Prism 5 (GraphPad Software). Data were presented as mean ± SD. Unpaired student *t* tests were used to test differences between spatial characteristics of different ROIs. *p* values <0.05 were considered statistically significant.

## Results

### High-resolution intravital imaging of the mouse brain vasculature is limited to a depth of 200 µm

**Fig. 1:**
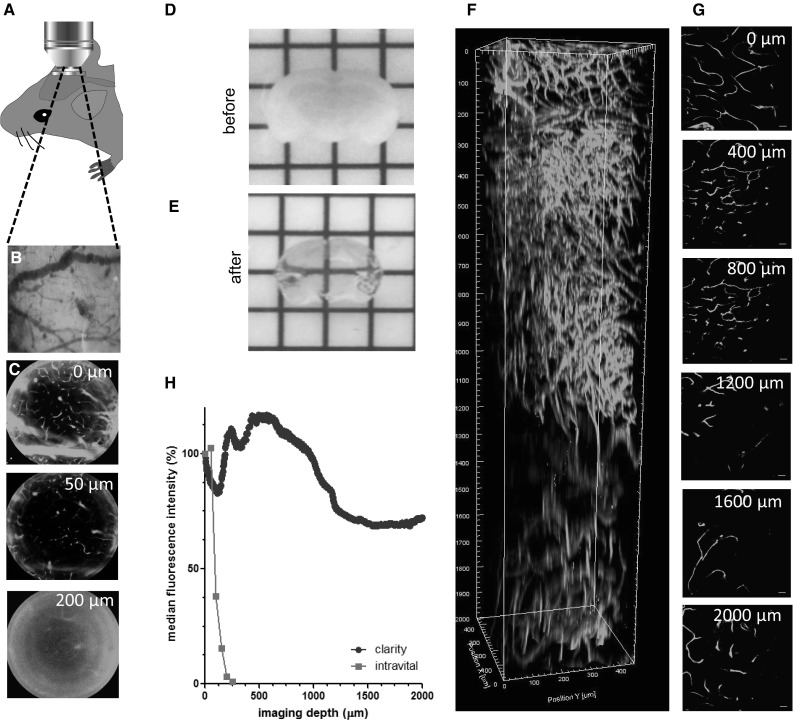
Intravital and *ex vivo* two-photon imaging of mouse cerebral microvasculature. **a** Schematic representation of intravital two-photon imaging setup. **b** Superficial blood vessels of the dura mater, observed through a cranial window. **c** 2D x–y plains obtained by intravital two-photon imaging of lectin-FITC-labeled cerebral mouse vasculature at 50-µm depth intervals (*n* = 1). *Scale bars* 100 µm. **d** 3-mm-thick slide of mouse brain, before optical clearing. **e** Transparent mouse brain after optical clearing with the iDISCO procedure. **f** Two-photon imaging of lectin-FITC-labeled brain tissue, cleared with the CLARITY procedure. Lateral view of a 3D reconstruction of composed of 1001 pictures, starting at the parietal cortical surface to 2000 µm below, see also Supplementary Movie S1. **g** 2D x–y plains of *ex vivo* two-photon imaging of vasculature in CLARITY-cleared mouse brain tissue at 400-µm depth intervals. *Scale bars* 100 µm. **h** Relative fluorescence intensity as a function of imaging depth for intravital 2-photon imaging (*n* = 1) and *ex vivo* two-photon imaging of CLARITY-cleared mouse cerebral microvasculature (*n* = 1). (Color figure online)

Intravital imaging of the mouse cerebral vasculature was performed by two-photon imaging through a cranial window as schematically shown in Fig. 1a. Through this window, the superficial blood vessels, which can be used for orientation, are easily recognized (Fig. 1b). Blood vessels, fluorescently stained with lectin^tomato^-FITC, were imaged at 50-µm intervals. Under these conditions, the maximal imaging depth was limited to 200 µm (Fig. 1c, h). Because this imaging depth was insufficient to image relevant locations deep into the brain, we decided to further explore *ex vivo* imaging methods for the visualization and quantification of the relation between tumor cells and the brain microenvironment, with a focus on the brain tumor vasculature.

### *Ex vivo* two-photon imaging of optically cleared brain tissue

In order to microscopically visualize regions throughout the entire mouse brain, optical clearing methods of mouse brain tissue were employed. Brains were stained and cleared by either the CLARITY [31] procedure or the methanol-based iDISCO method [32]. Brain slides of 3–5 mm thickness were processed to render transparent tissue, as shown for the iDISCO procedure in Figs. 1d, e. Whereas iDISCO leads to a stable shrinkage of approximately 1.2× of the original tissue diameter (Fig. 1e), the CLARITY procedure resulted in an expansion of approximately 1.5× the tissue size. This tissue expansion continued after prolonged storage of the tissue in 80% glycerol, which complicates re-analysis of CLARITY-cleared tissues, whereas tissues which were previously cleared and stained could be re-imaged for subsequent analyses months later (data not shown). Within the parietal cortex, lectin^tomato^-stained microvessels could be imaged by 2-photon microscopy down to a depth of 2000 µm after CLARITY optical clearing (Fig. 1f–h, Supplementary Fig. S1 and Supplementary Movie S1). In contrast to the rapid loss of signal intensity at increasing depth during intravital imaging, fluorescent signal intensity was well maintained in optically cleared tissues. In optically cleared tissues, signal intensity remained above 65% of superficial signal intensity along the complete working distance of the lens of 2000 µm (Fig. 1h).

### Computational modeling of vasculature in healthy mouse brain tissue

**Fig. 2:**
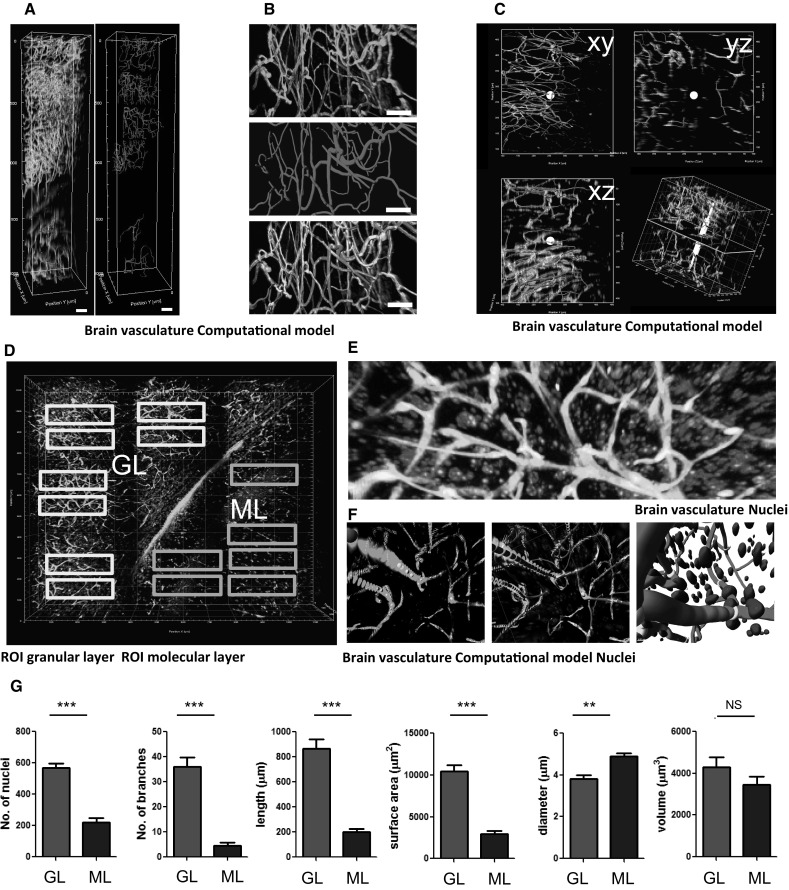
3D representation and computational reconstruction of cerebral microvasculature and surrounding cells. **a** Computational modeling (*red*) of 3D cerebral vasculature fluorescently labeled with lectin^tomato^-DyLight488 (*green*). *Scale bars* 100 µm. **b** *Upper panel*: fluorescently labeled vasculature (*green*), *mid panel*: computational reconstruction modeled with Imaris filaments tool (*red*), *bottom*: overlay of the fluorescent signal with the computational reconstruction. *Scale bar* 25 µm. **c** 2D orthogonal images of computational reconstruction (*red*) and fluorescent signal (*green*). **d** Areas in the granular layer of the cerebellum (gl, *green rectangles*) and the molecular layer of the cerebellum (ml, *blue rectangles*) are selected for quantification. *Scale bar* 100 µm. **e** Details of a granular layer region, showing the spatial relation of the nuclei (*blue*) to the vessels (*green*). *Scale bar* 25 µm. **f** Computational modeling of vasculature (*red*) and nuclei (*blue*) of the microvascular and surrounding cells. *Scale bars* 10 µm. **g** Quantification of number of nuclei, number of vessel branches, total vessel length, total surface area, mean vessel diameter and total vessel volume in the granular layer and molecular layer regions of the cerebellar cortex. All quantifications are normalized to a volume of 10^6^ µm^3^. **p < 0.01, ***p < 0.001, *t* test. (Color figure online)

Understanding the relation between glioblastoma cells and the vasculature would benefit from quantification of different aspects of their relationship. With the use of healthy mouse brain tissue, a workflow for the quantification of the vasculature was developed (Supplementary Fig. S2). In a stack of lectin^tomato^-FITC-stained vessels (Fig. 2a, green), different regions of interest (ROIs) were selected for computational modeling (Fig. 2a, red regions). Fluorescently stained blood vessels (Fig. 2b, green, upper panel) were converted to computationally modeled vessels (Fig. 2b, red, middle panel). Because these computational models were unbiased, i.e., without manual postprocessing, the overlay of the modeled vessels showed a high degree of concordance, but was not perfect. (Figure 2b overlay, bottom panel). By using 2D orthogonal planes, we estimated the concordance of the fluorescently labeled vasculature with the computational modeled vasculature to be approximately 80% (Fig. 2c, Supplementary Fig. S4). Next, the combination of lectin^tomato^-FITC-labeled microvasculature and counterstained nuclei (DAPI) was imaged using two-photon microscopy (Fig. 2d). These large, tiled image files show a striking vignetting at the borders of each acquired imaging field. This vignetting is due to the fact that standard water immersion or air objectives—which are not corrected for the high refractive indexes of the embedding medium—were used for the acquisition. Because this could potentially result in artifacts in the stitching and consequent quantification, selected ROIs were centered within the tiles. ROIs were selected within the granular layer (GL, green rectangles) and in the molecular layer (ML, blue rectangles) of the cerebellum (Fig. 2d). The obtained 3D stack with lectin^tomato^-stained microvessels and DAPI-stained nuclei enabled detailed fluorescently imaging (Fig. 2e) and allowed computational modeling (Fig. 2f). After such computational modeling, the number of DAPI-stained nuclei, and quantitative data of the vasculature, such as number of branches, total vessel length, volume, area, and diameter of single segments per volume of 100 × 100 × 100 µm (=10^6^ µm^3^), were calculated. These analyses revealed large differences in the vasculature of the GL versus the ML of the cerebellum (Fig. 2g, Supplementary Movie S2). These observed differences corroborate findings of previous studies reporting that, within different brain regions, large differences in vasculature are present [35], thus emphasizing that only identical, anatomically well-defined, areas should be compared to evaluate spatial relations between cells and their brain microenvironment.

### The lectin^tomato^-staining pattern of the microvasculature is not affected by GBM cells

**Fig. 3:**
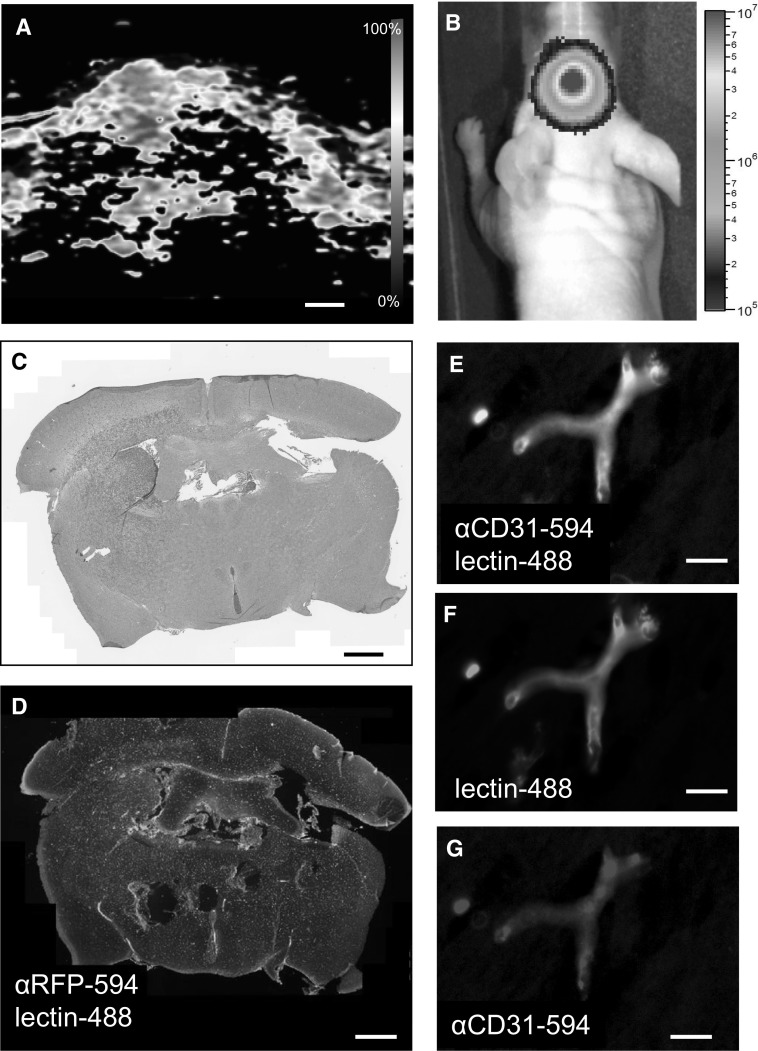
Lectin^tomato^ binding to the microvasculature in the presence of tumor cells. **a** Photoacoustic coronal image of an intracranial GBM8-FM tumor with highly oxygenated, vascular regions indicated in *red*. **b** BLI image showing the presence of the GBM8-FM tumor. **c** Hematoxylin/eosin-stained section of the same tumor as depicted in **a**, **b** showing the association between highly vascularized/oxygenated areas and the presence of tumor. **d** Brain tissue with GBM8-FM tumor stained with lectin^tomato^-DyLight488 (*green*)-directed against blood vessels, and with an anti-RFP-AlexaFluor594 antibody directed against the GBM8-FM cells. **e**–**g** Details of (**d**) showing that in the presence of tumor, lectin^tomato^ (*green*) and anti-CD31 antibody (*red*) stain the same microvessels. *Scale bars* a, **c**, **d** 700 µm; *Scale bars* **e**, **f**, **g** 10 µm. (Color figure online)

Because tumor cells are known to alter the endothelial glycocalyx [36], we confirmed the binding of lectin^tomato^ to blood vessels in tumor-containing tissue. Brain tumors were induced by orthotopic xenograft transplantation of human GBM8-FM cells into nude mice. First, the presence of a GBM8-FM tumor was detected by photoacoustic imaging, which revealed increased oxygenation levels in the tumor areas, indicative for a change in the vasculature (Fig. 3a). Tumor growth was confirmed by bioluminescence imaging (Fig. 3b), and H&E staining of the brain clearly indicated the presence of tumor in the striatum (Fig. 3c). Furthermore, tumor was easily identified on tissue slides stained with lectin^tomato^ and anti-RFP antibody, which stains the GBM8-FM cells (Fig. 3d). Combined staining with lectin^tomato^ and anti-CD31 (Fig. 3e–g) demonstrated overlay of lectin^tomato^-stained and CD31-stained microvasculature, indicating that tumor growth does not significantly influence the lectin-binding pattern of brain microvasculature.

### Computational modeling of the GBM vasculature in mouse brain

**Fig. 4:**
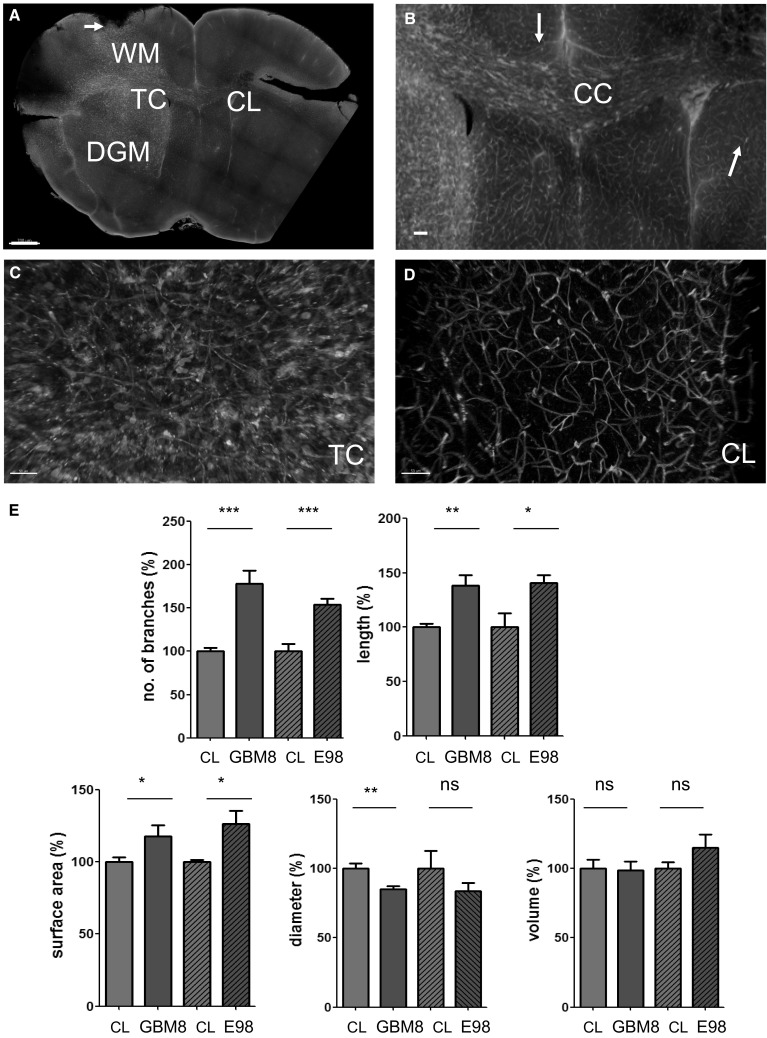
Microvascular reconstruction in GBM infiltrated areas. **a** Typical example of a diffuse, invasively growing GBM8-FM tumor (anti-RFP-Ax594, *red*) with its microvasculature (lectin^tomato^-DyLight488, *green*). Tumor cells have migrated into the healthy brain parenchyma, away from the tumor core (TC) into the contralateral (CL) hemisphere, into the deep gray matter (DGM), and into the subarachnoid space (*arrows*). **b** Details of (**a**) showing the abundant presence of GBM cells in the corpus callosum (CC). **c** Details of the TC, with tumor cells (*red*) in close proximity to blood vessels (*green*). **d** Details of (**a**) showing distantly migrated solitary tumor cells (*red*) in close proximity to blood vessels (*green*). **e** Quantification of number of vessel branches, vessel length, surface, diameter and vessel volume in the tumor core of GBM8 tumors (*n* = 7) and E98 (*n* = 2) tumors. Values are normalized to comparable control areas (CL) within the same brain where no evident tumor formation was detected. *p < 0.05, **p < 0.01, ***p < 0.001, *t* test. *Scale bars* **a** 700 µm; **b** 250 µm; **c**, **d** 50 µm. (Color figure online)

As the iDISCO method allowed easy processing of already formalin-fixed materials, this method was employed for optical clearing of the formalin-fixed tumor bearing brain tissues. The optical clearing procedures were performed with GBM8-FM xenografted brain tissues which were fixed for less than 2 months and with E98-FM xenografted brain tissues which were fixed for over 2 years. Tissue stained for tumor cells (anti-RFP-Ax594, red) and vasculature (lectin^tomato^-DL488, green) revealed the presence of large, diffuse infiltrative tumors in the GBM8-FM glioblastoma xenograft models (Fig. 4a, Supplementary Movie S3). GBM cells have migrated away from the tumor core (TC) into the deep gray matter (DGM) and white (WM) matter areas, and even to the contralateral side (CL). Also, migration into the subarachnoid space was observed (arrow). Figure 4b shows that large number of GBM cells migrated collectively through white matter tracts such as the corpus callosum (CC). However, both in the hemisphere where the tumor was transplanted, as in the contralateral hemisphere, solitary cells can be identified, especially in close proximity to blood vessels (arrows). In the tumor core, changes in the vasculature were shown (Fig. 4c) as compared to the vasculature in the corresponding region on the contralateral side (Fig. 4d). Striking is the binding of lectin^tomato^ on cell-like structures in the tumor core (Fig. 4c), where these lectin-stained cells are virtually absent in the contralateral side (Fig. 4d). Computational modeling of the vasculature in these lectin-positive cell-rich areas was feasible, because the filament tracer detects tube-like structures (Supplemental Fig. S6). Quantifications of GBM8 and E98 tumors was performed by comparing contralateral brain areas within the same brain. The control area was normalized to 100% to allow a pair-wise comparison of the tumor versus control area. The quantifications revealed an increase in the number of vessel branches, increase in total vessel length, and an increase in surface area of these vessels within the tumor regions. The mean vessel diameter was slightly lower in the tumor core as compared to the same region of the contralateral side, resulting in comparable vessel volume in both areas for both the GBM8 tumor and the E98FM tumor (Fig. 4e).

### GBM cell distribution in the brain microenvironment

**Fig. 5:**
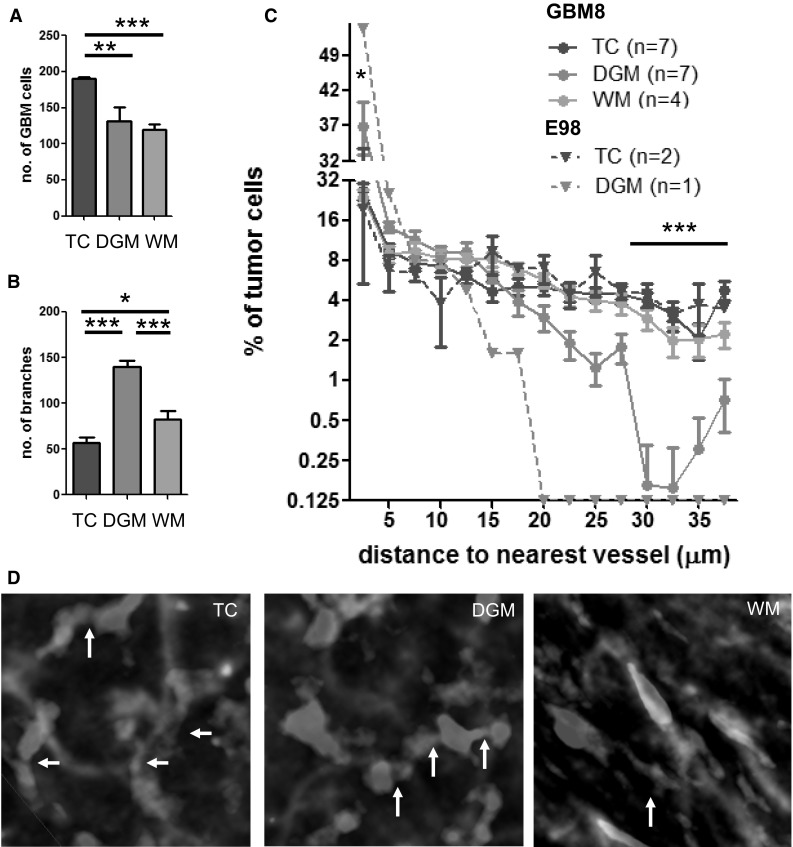
Topographical characteristics of GBM cells distant from the tumor core. **a** GBM cell density (per volume of 10^6^ µm^3^) was calculated in the tumor core (TC, *n* = 7), and invasive fronts within the *deep gray* matter (DGM, *n* = 7) and white matter (WM, *n* = 4) of GBM8 tumors. **b** Number of vessel branches (per volume of 10^6^ µm^3^ in the three aforementioned areas in GBM8 xenografted brains. **c** Proportion of GBM8 and E98 cells at indicated distance (µm) of the nearest vessel at distribution intervals of 2.5 µm. **d** GBM8 cells are interconnected via cell processes (*arrows*). *p < 0.05, **p < 0.01, ***p < 0.001, *t* test. *Scale bars* 10 µm. Samples were cleared with iDISCO. (Color figure online)

We quantified the number of GBM cells in three different anatomical areas (TC, DGM, WM), as illustrated in Fig. 4a. The number of GBM8 cells per 10^6^ µm^3^ in the tumor core was significantly higher as compared to the infiltrative fronts in both the deep gray matter and the white matter regions (Fig. 5a). Similar as shown in Fig. 2g, large differences in vessel characteristics were observed between distinct anatomical regions, as shown for the number of vessel branches (Fig. 5b), and other parameters (Supplementary Fig. S7).

Next, we calculated the distance of the GBM8 tumor cells to the microvessels in the three anatomical areas. In the observed E98-FM tumor xenografts, infiltration was less pronounced as compared to the GBM8 tumors (Supplementary Fig. 5), which limited the quantitation of the E98 tumor cells to the tumor core and the gray matter areas. In the tumor core, 29.8% of the GBM8 tumor cells were located at a distance of >20 µm from the nearest blood vessel. Tumor cells in the infiltrative front within the deep gray matter were more closely located to the blood vessels; in this gray matter area, only 9.5% of the GBM8 tumor cells was located at a distance of >20 µm from their nearest blood vessel (Fig. 5c). The E98 tumor cells were even more closely associated with the vasculature, with >99% of the invasive GBM cells in the deep gray matter located at a distance of <20 µm from the nearest blood vessel (Fig. 5c). These results may suggest some preference for perivascular migration of GBM cells in the deep gray matter, whereas in the tumor core alternative migration routes, such as through the brain parenchyma, are used. An alternative explanation is that proliferation of the GBM cells in the tumor core has resulted in an expansion of the intervascular compartment, thus resulting in larger distances of the tumor cells from the microvasculature. Interestingly, in the white matter regions, the distance distribution of the GBM cells to the nearest blood vessels was comparable to the tumor core regions, suggesting that tumor cell migration in the white matter area does not solely depend on blood vessel co-option, but may also occur via white matter tracts. Another striking observation is the presence of a subset of tumor cells that are interconnected by cellular processes similar as the multicellular networks as postulated by Osswald et al. [37]. Groups of approximately 4–6 cells were interconnected by tubular, ‘dendrite-like’ structures (Fig. 5d, arrows). These results demonstrate that optically cleared tissues can be used to detect subtle changes in the brain tumor microvasculature, and allow visualization and quantitation of GBM cells, and quantitation of the relative position of GBM cells to the brain microvasculature. The proposed workflow (Supplementary Fig. S2) offers an attractive additional method for 3D histology (Supplementary Movie S3), complementary to traditional histological methods.

## Discussion

In the present study, we show the use of optically cleared brain tissues to allow visualization of cerebral microvasculature and the organization of nuclei or tumor cells in the brain tumor microenvironment. For this, we developed an effective workflow, which was explored to quantify subtle changes in the vasculature, and moreover, to visualize and measure the topographical relationship between GBM cells and their microenvironment in an invasive orthotopic GBM model in mice. Transparent adult mouse brain tissues were prepared with the CLARITY protocol or the iDISCO procedure. Both methods allowed detailed optical imaging and 3D reconstruction of the microvasculature and distribution of cells or nuclei. Computational modeling allowed quantitative analysis, showing differences in the GBM cell distribution in distinct regions of the brain.

Intravital imaging was not suitable to image relevant regions of intracranial tumor due to its limited imaging depth and small fields of view. Therefore, we extended our methods to study the tumor microenvironment to *ex vivo* 3D imaging of transparent brain tissues. *Ex vivo* 3D whole-tissue imaging has become a maturing field of research [38], and many tissue-clearing protocols have been developed recently. Most of these clearing protocols focus on improvement of fluorescence signal intensity because conventional clearing protocols caused quenching of fluorescence signal. CLARITY and iDISCO procedures have been reported to be compatible with fluorescently stained tissues [18, 20, 31, 32]. Both procedures remove the highly abundant lipids in myelin-rich brain tissue [39], which is an important step to obtain optically transparent tissues, but also improves antibody penetration. Improvement of antibody penetration is important because immunofluorescent staining of large tissues is still a challenge [40, 41]. Furthermore, we noticed that certain antibodies require further optimization for staining of large tissues. For example, anti-vimentin staining, which was successfully used to visualize E98FM tumor cells on traditional 5-µm sections [42], did not result in detection of tumor cells in our iDISCO processed samples, and the CD31 staining was hampered by low signal-to-noise ratio (data not shown). For the use of antibodies for additional markers, optimization steps such as titration of the antibody concentrations, and tissue pretreatment, needs to be validated for each antibody [18]. Besides the use of antibodies to obtain clear structure information, these antibodies could also be validated for their use to provide functional information. These could include the staining of IgG leakiness [43] or the use of other proteins which leak out of the vasculature after BBB disruption. To ensure complete and consistent tissue staining, we limited the thickness of the tissue slides to approximately 3–5 mm and explored the use of lectins, which have a low molecular weight (71 kD), as compared to antibodies (150 kD). The used lectin, isolated from Lycopersiconesculentum (*tomato)*, has a high affinity for glycan epitopes on endothelial cells [43–45], and on ramified and activated microglia [44, 46], which might explain the observed binding of lectin^tomato^ to non-endothelial cells within the brain tumor. In addition, to quantify the cerebral vasculature and surrounding cells, we have extended the possibilities of 3D visualization of cleared brain tissue by the addition of computational modeling with the commercial software package Imaris.

For imaging of optically cleared tissues, numerous microscopical techniques have been developed, including two-photon imaging, light sheet microscopy and confocal imaging, which all have their own advantages and limitations (Supplementary Fig. S8). All used microscopes in this manuscript were equipped with standard air or water lenses. Hence, vignetting is visible on the edges of all acquired images which leads to artifacts in the stitching of the tiled images. To prevent these artifacts, special lenses tailored for CLARITY are developed which could further improve the quality of tiled images [47].

**Fig. 6:**
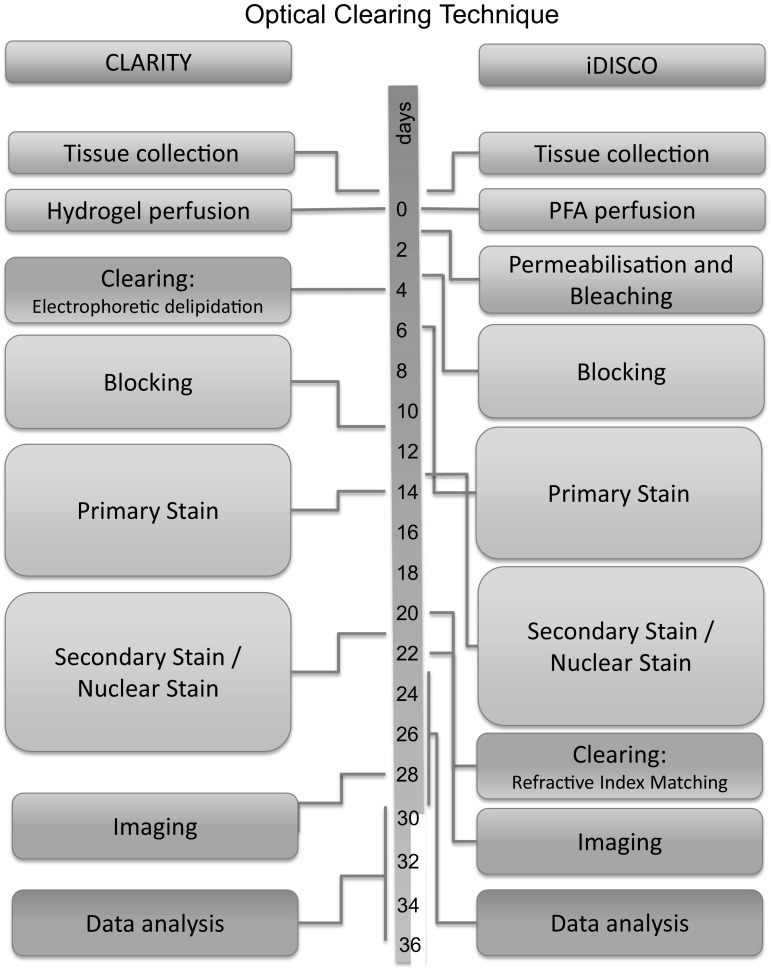
Time lines for the process from tissue collection to data analysis. This figure represents typical timelines for tissue of 5 mm thickness, which requires approximately one week for each antibody or lectin staining. Because the incubation steps are not very labor-intensive, it is feasible to process several tissues simultaneously. (Color figure online)

The whole process, from tissue collection to sample preparation, image acquisition, reconstruction, postprocessing, analysis and 3D image acquisition, takes approximately one month for both the CLARITY procedure and the iDISCO procedures (Fig. 6). This processing time is highly influenced by the thickness of the tissue as diffusion of antibodies through the tissue is a time-consuming process. However, although these methods to prepare tissue for optical sectioning are still time-consuming, they are not as labor-intensive as compared to traditional methods of mechanically, histological sectioning.

GBM is still incurable, partly because of its invasive character, but also because interactions with its microenvironment and intra-tumoral communication. The invasive character of GBM was already thoroughly described in the 1930s. In that work, four pathways of glioma cell invasion were recognized: through the brain parenchyma, along preexisting blood vessels, through white matter tracts and via the subarachnoid space [48, 49]. The distribution of GBM cells we observed is fully compatible with those four migratory patterns. We hypothesized that these migration patterns will be reflected by the distance of the tumor cells to the microvasculature. Indeed, we found that in gray areas, tumor cells migrated mainly in close proximity of the vasculature. In contrast, in the white matter a bigger proportion of the GBM cells were located at a larger distance from the microvasculature, suggestive for migration following the tracts of the white matter. In GBM, intra-tumoral communication may take place via secreted factors, including exosomes [50], or by direct cell–cell contact of the tumor cells via an interconnected GBM cell network [37, 51–53]. In line with this, we observed the presence of interconnected GBM cells, similar as suggested by Osswald et al. [51]. These cell clusters were present in the tumor core, but also in the infiltrative fronts in the deep gray matter and the white matter.

In conclusion, we demonstrate the use of optically cleared, transparent tissues to study in 3D the architecture of the brain microvasculature as well as the topographical relationship of GBM cells to the microvasculature. Detailed 3D visualization and quantitation revealed differences in organization of tumor cells relative to the vasculature in gray matter regions versus white matter regions, and patterns of GBM cell networks collectively invading the brain parenchyma. This deep-fluorescence imaging and 3D quantitative approach opens new avenues to study the pathobiology of brain cancer cells and their microenvironment.

## Importance of the study

Studying brain tumors in relation to the brain microenvironment requires in-depth 3D imaging techniques. The use of optically cleared, transparent tissues may enable such studies and supplement currently used laborious methods of mechanically slicing and aligning of tissue slices. We show that routes of glioblastoma invasion can be studied in transparent mouse brain tissues. By quantitation of the topological orientation of GBM cells to the microvasculature we show the feasibility to visualize in high resolution (organelle level) glioblastoma cells in the context of the brain microenvironment. We identified GBM cells co-opting the brain vasculature, GBM cells invading along white matter tracts, and groups of infiltrated GBM cells interconnected via ‘dendrite-like’ structures indicative of collective invasion of the brain microenvironment.

## Electronic supplementary material

Below is the link to the electronic supplementary material.

Supplementary material 1 (AVI 21044 kb)

Supplementary material 2 (AVI 51045 kb)

Supplementary material 3 (MP4 25669 kb)

Supplementary material 4 (PPTX 4050 kb)
