# Supplementary material for: Optical clearing and fluorescence deep-tissue imaging for 3D quantitative analysis of the brain tumor microenvironment
Source: Angiogenesis. 2017 Jul 11;20(4):533–46. doi: 10.1007/s10456-017-9565-6 (PMC5660146; doi:10.1007/s10456-017-9565-6)
Supplement: Supplementary file 4 — Supplementary material 4 (PPTX 4050 kb) [file 10456_2017_9565_MOESM4_ESM.pptx]

## Slide 1
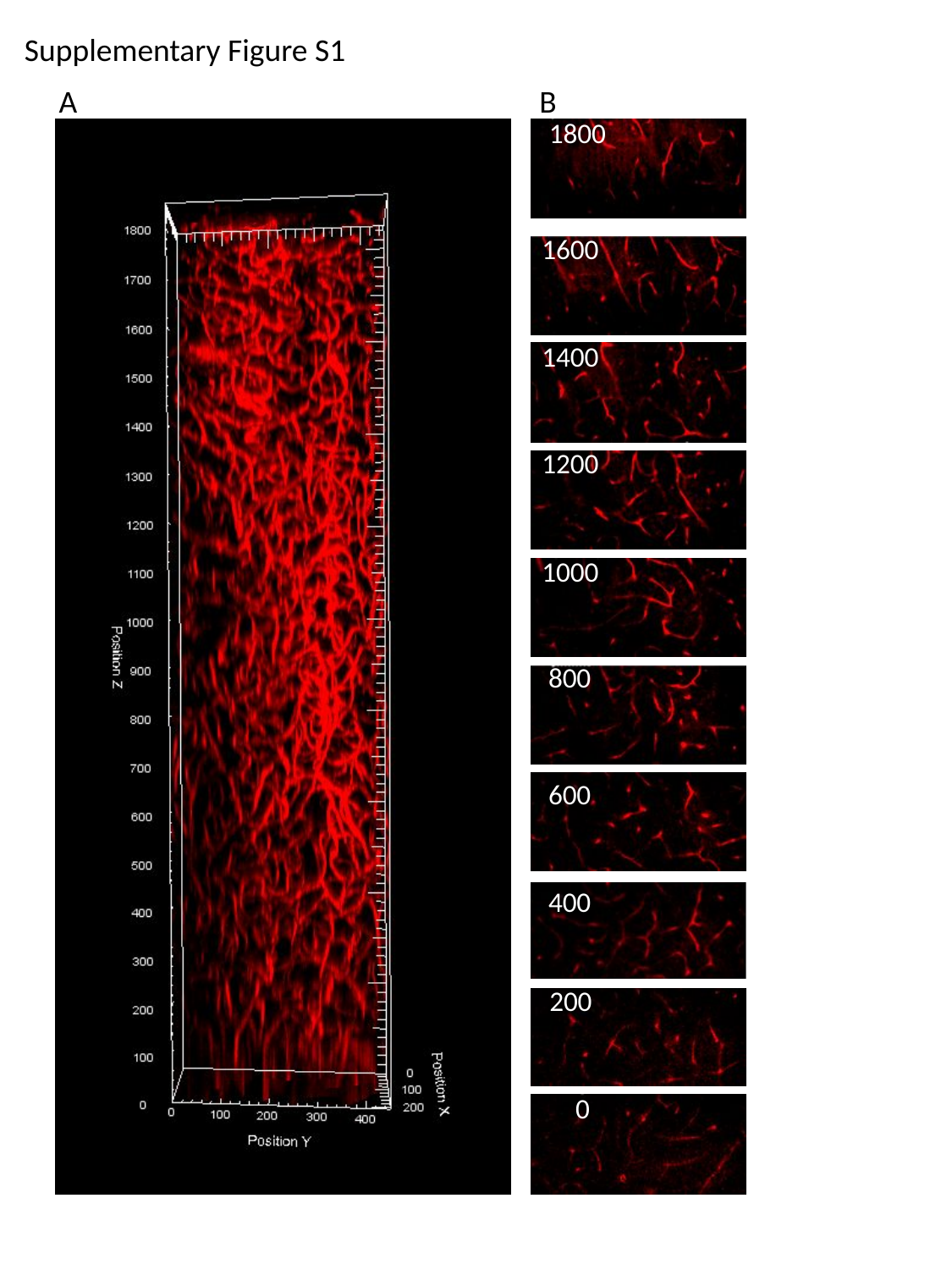

Supplementary Figure S1
A
B
1800
1600
1400
1200
1000
800
600
400
200
0

## Slide 2
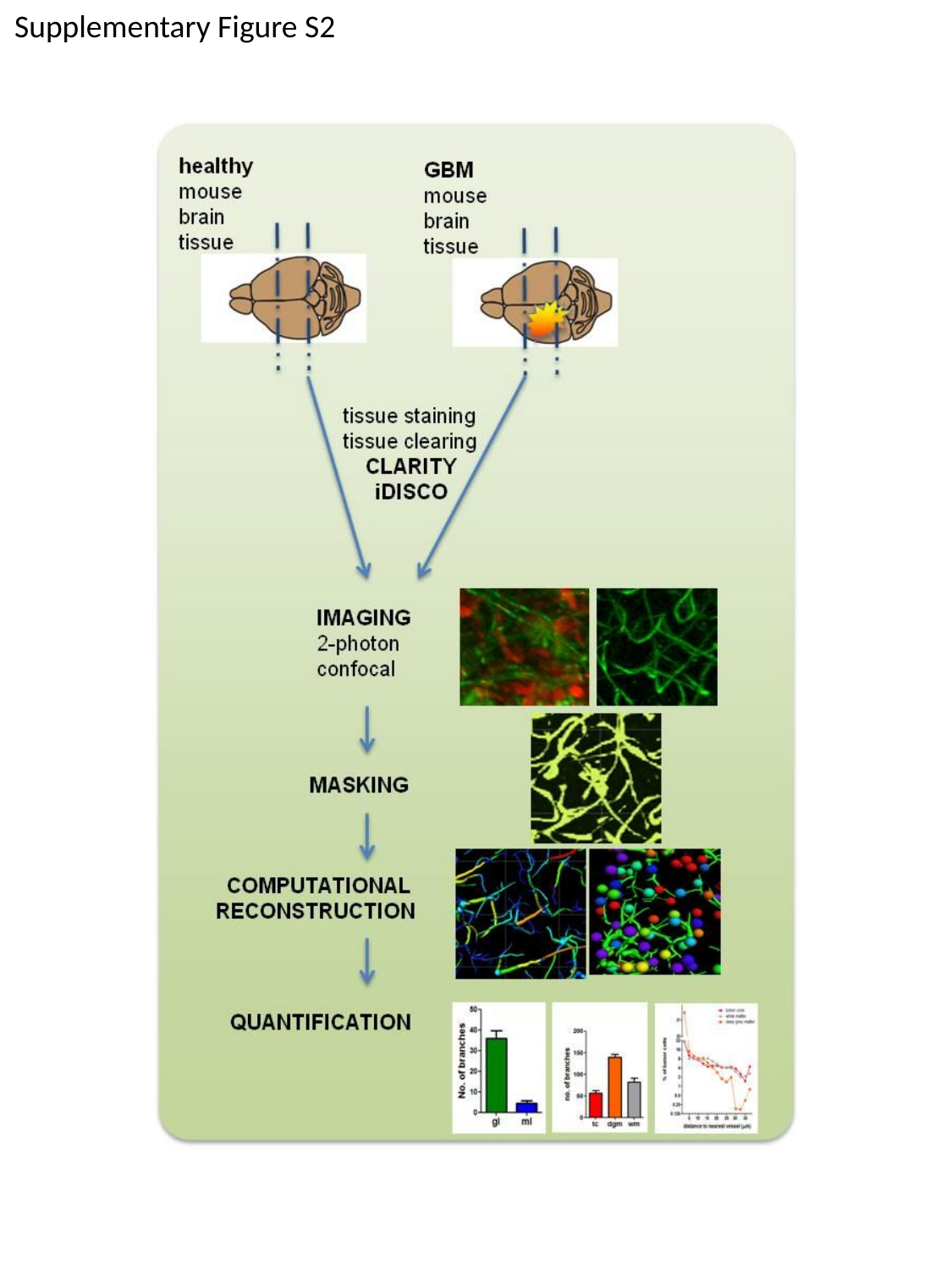

Supplementary Figure S2

## Slide 3
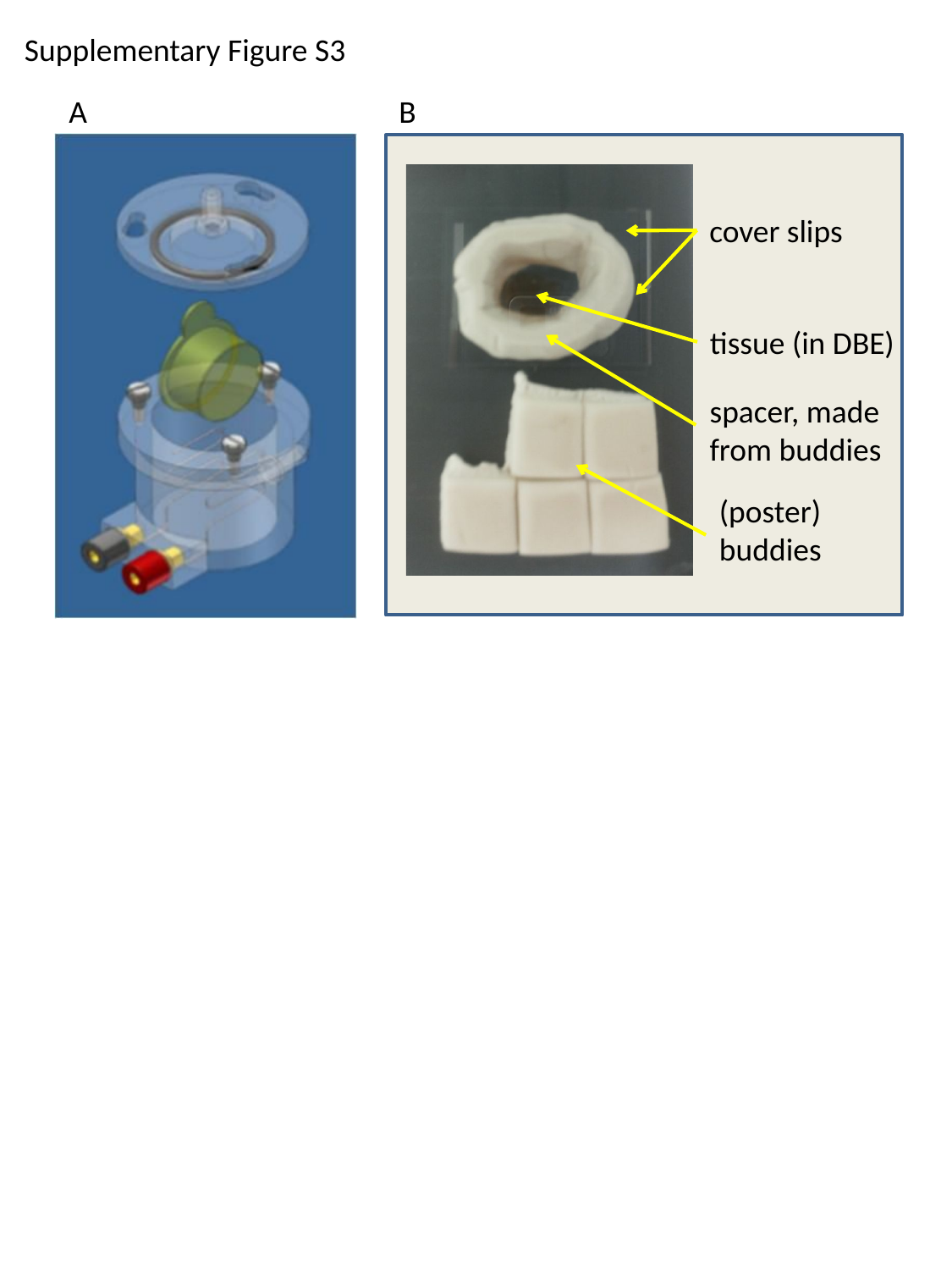

Supplementary Figure S3
A
B
cover slips
tissue (in DBE)
spacer, made
from buddies
(poster)
buddies

## Slide 4
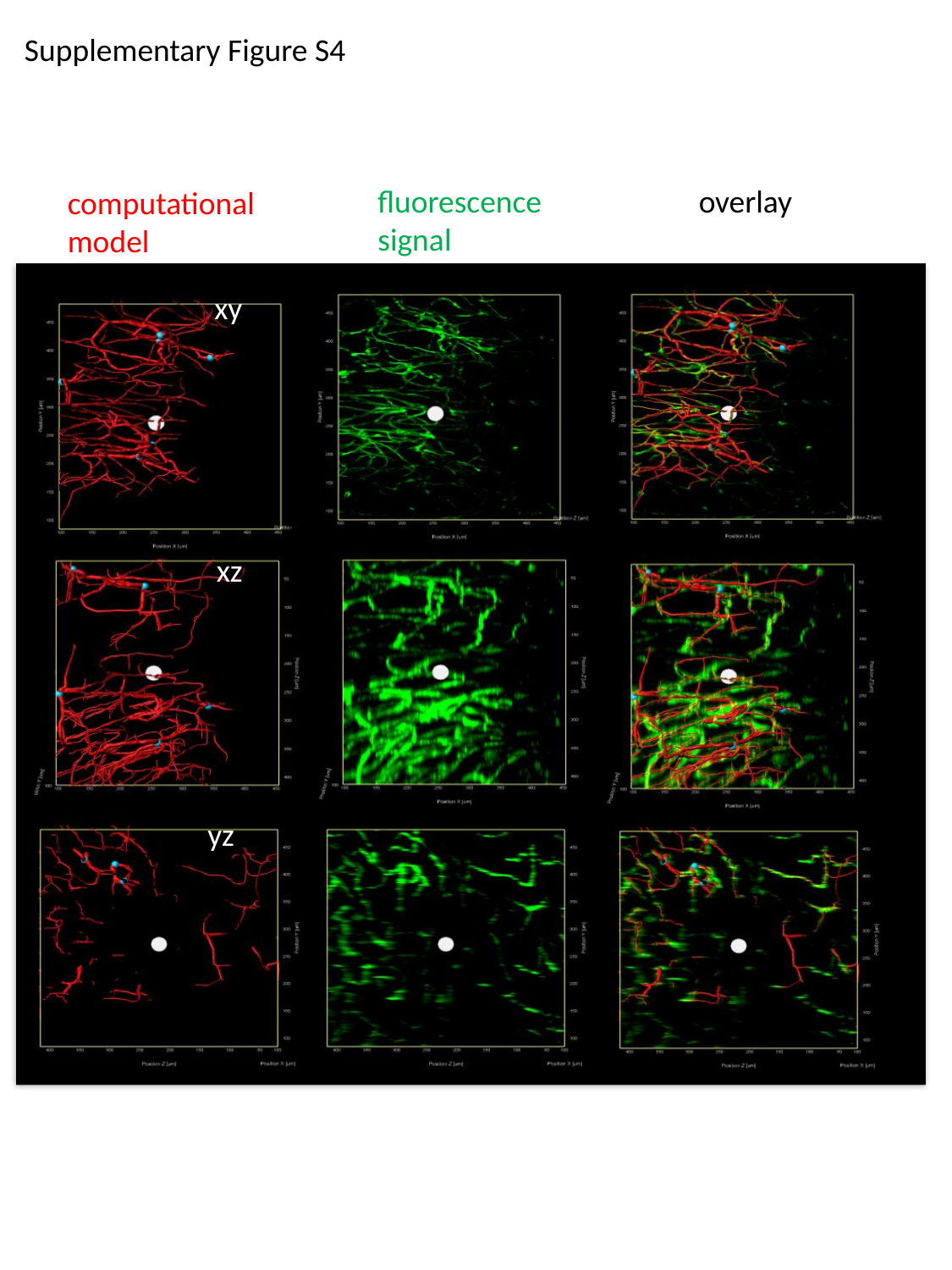

Supplementary Figure S4
fluorescence
signal
overlay
computational
model
xy
xz
yz

## Slide 5
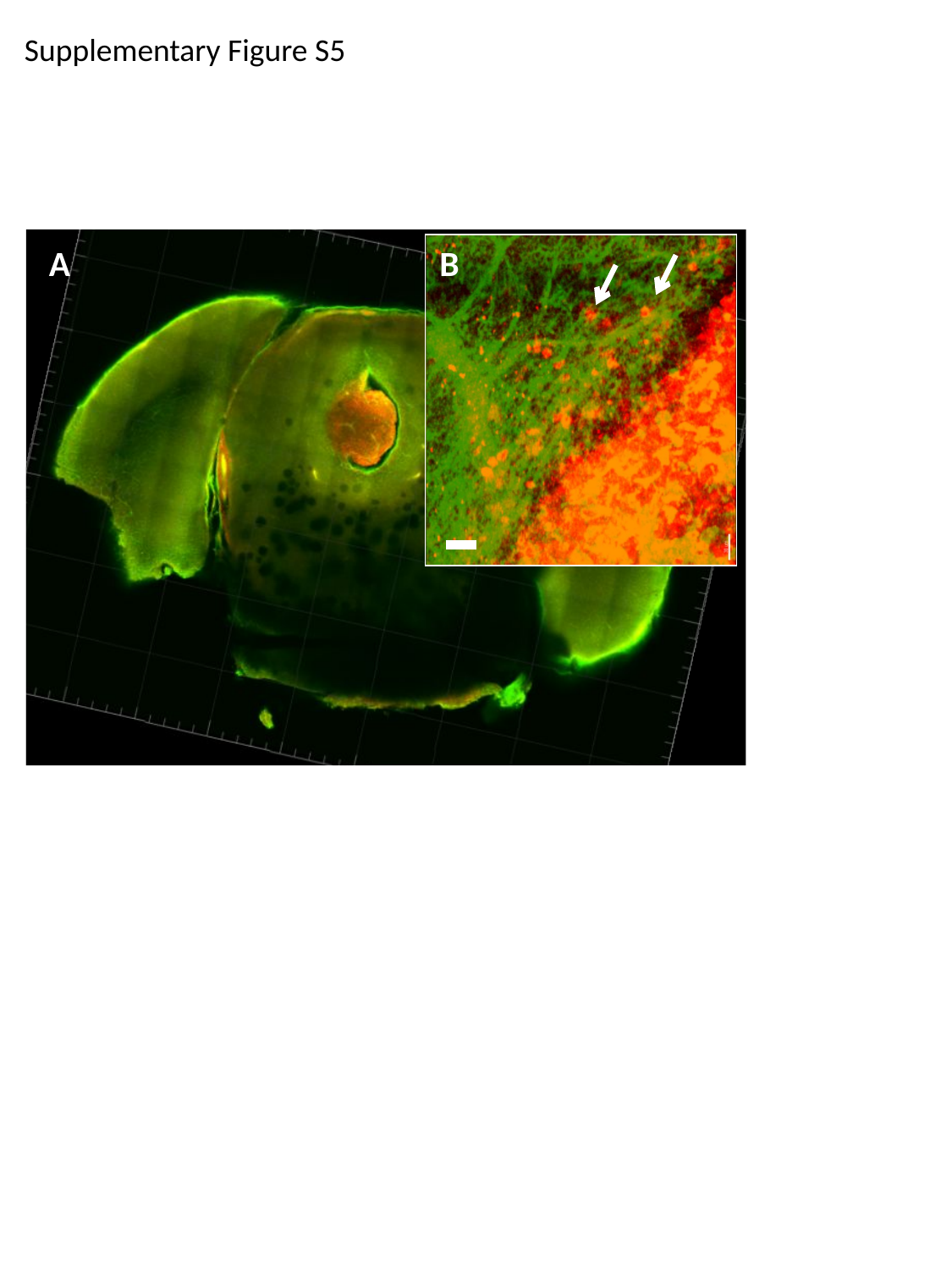

Supplementary Figure S5
A
B

## Slide 6
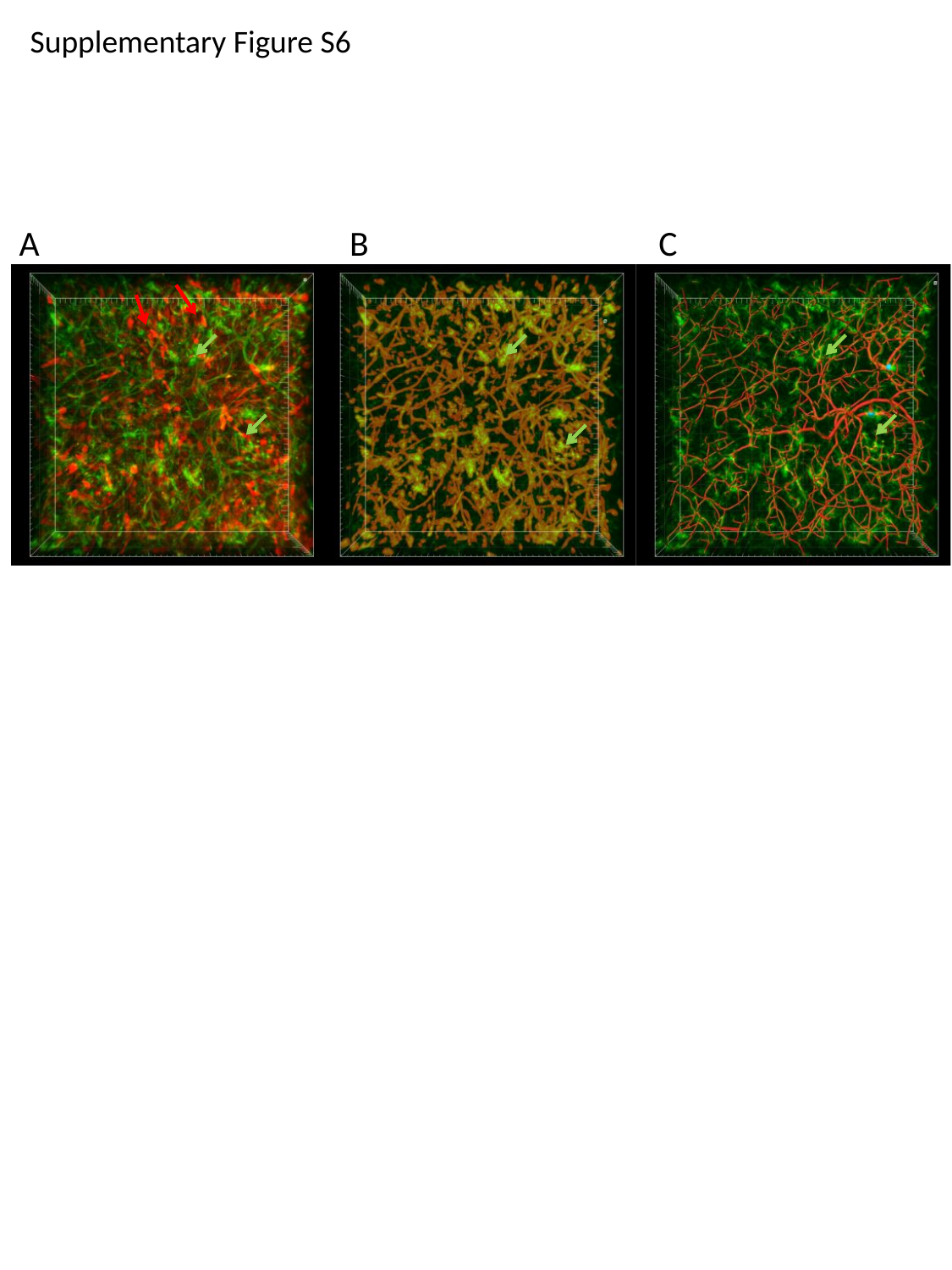

Supplementary Figure S6
A
B
C

## Slide 7
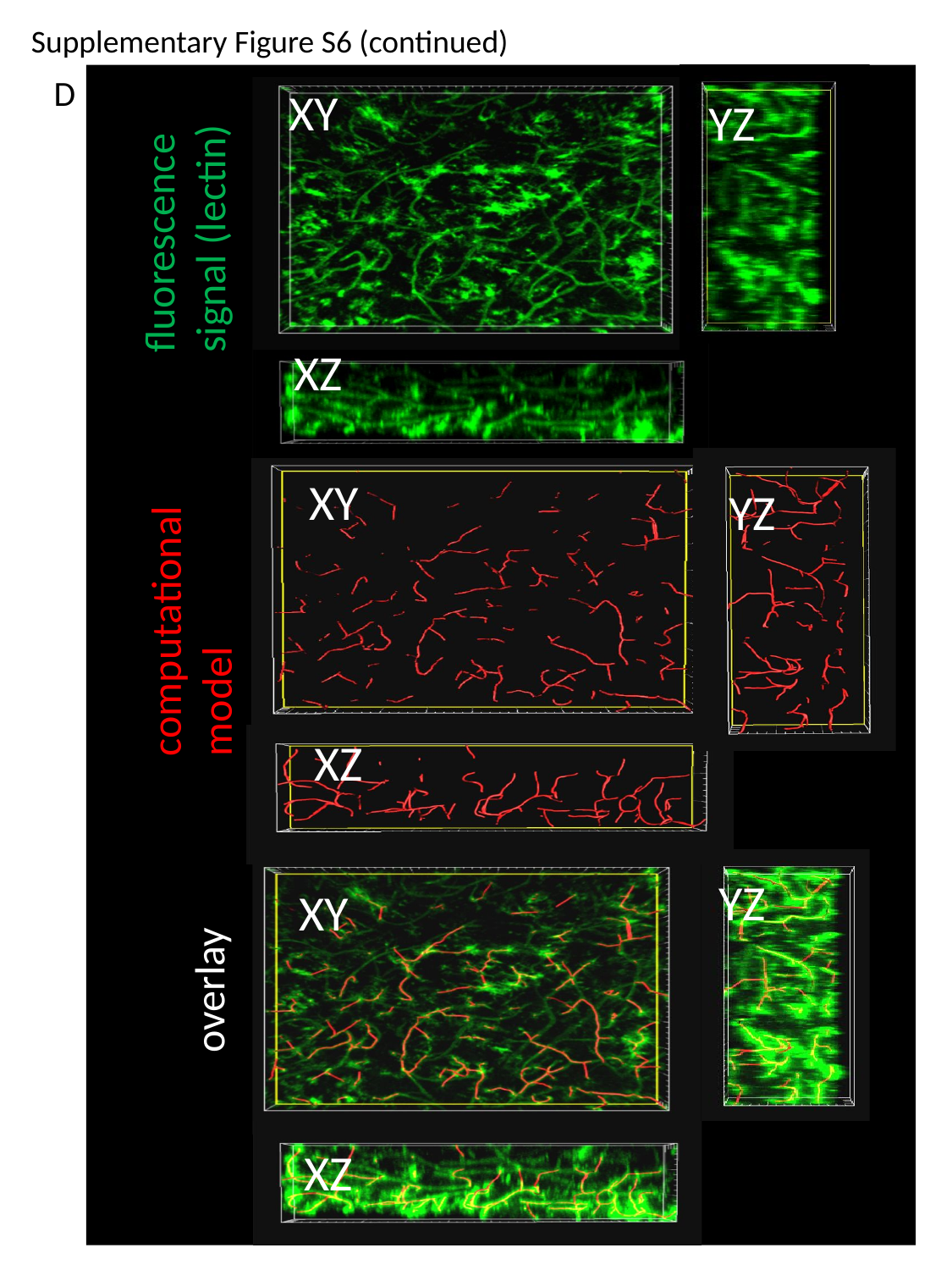

Supplementary Figure S6 (continued)
D
XY
YZ
fluorescence
signal (lectin)
XZ
XY
YZ
computational
model
XZ
YZ
XY
overlay
XZ

## Slide 8
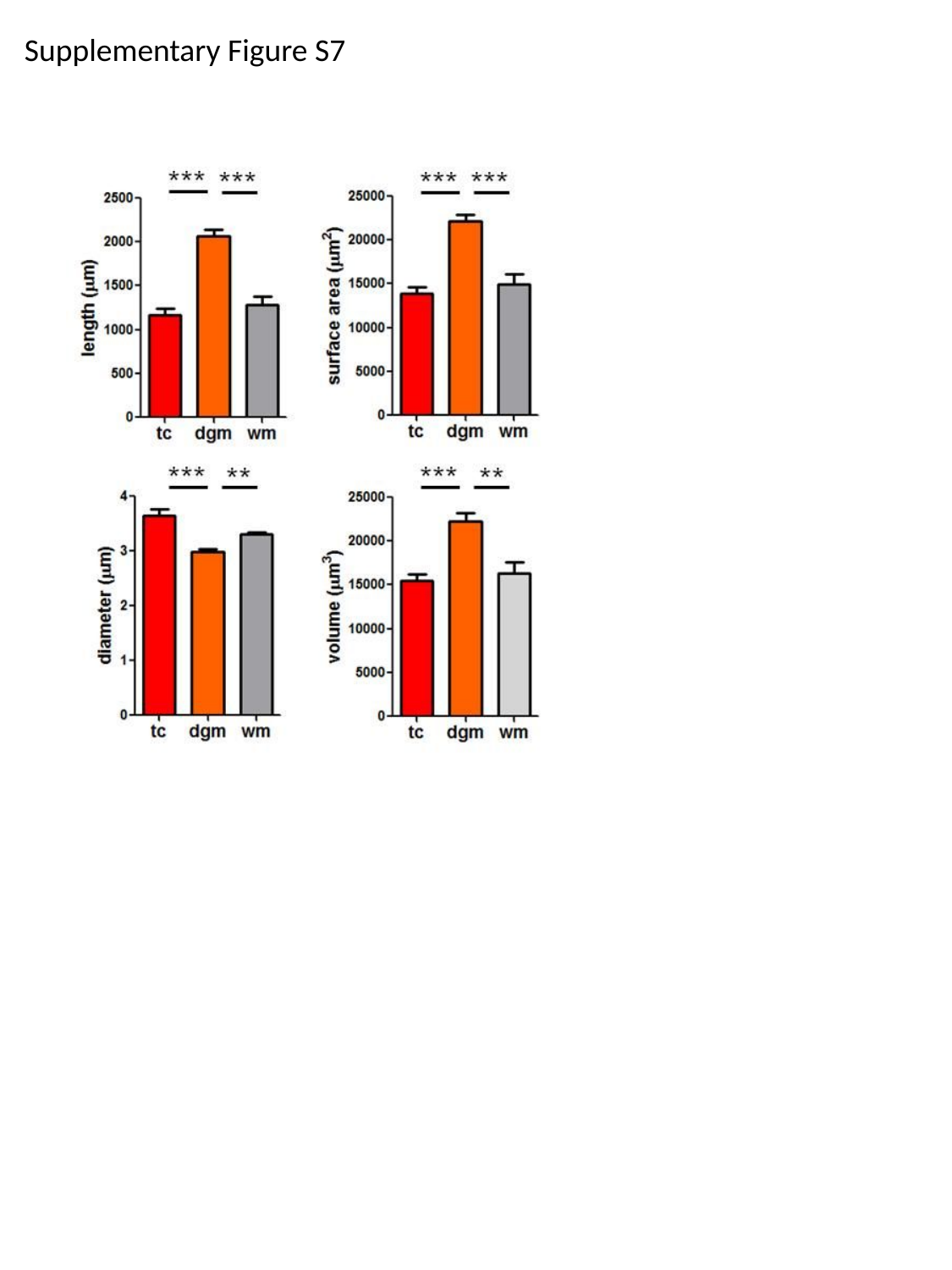

Supplementary Figure S7

## Slide 9
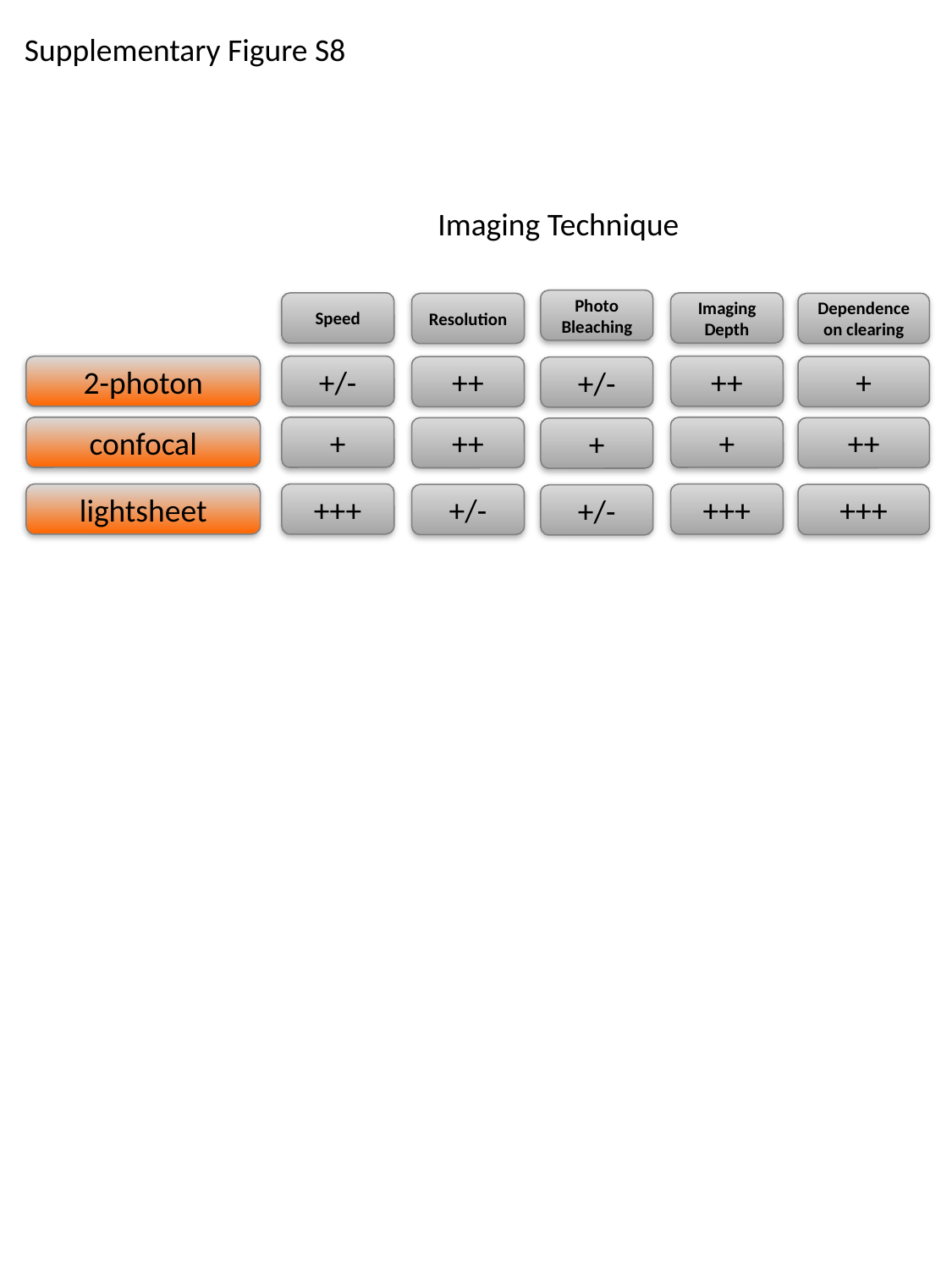

Supplementary Figure S8
Imaging Technique
Photo
Bleaching
Imaging
Depth
Speed
Resolution
Dependence on clearing
++
+/-
2-photon
++
+
+/-
+
confocal
+
++
++
+
+++
+++
lightsheet
+/-
+++
+/-
